# Human microRNA (miR-20b-5p) modulates Alzheimer’s disease pathways and neuronal function, and a specific polymorphism close to the *MIR20B* gene influences Alzheimer’s biomarkers

**DOI:** 10.1038/s41380-021-01351-3

**Published:** 2022-01-27

**Authors:** Ruizhi Wang, Nipun Chopra, Kwangsik Nho, Bryan Maloney, Alexander G. Obukhov, Peter T. Nelson, Scott E. Counts, Debomoy K. Lahiri

**Affiliations:** 1 Laboratory of Molecular Neurogenetics, Department of Psychiatry, Indiana Alzheimer’s Disease Research Center, Indiana University School of Medicine, Indianapolis, IN 46202 USA; 2 Radiology, Indiana University School of Medicine, Indianapolis, IN 46202 USA; 3 Anatomy, Cell Biology & Physiology, Indiana University School of Medicine, Indianapolis, IN 46202 USA; 4 Sanders-Brown Center on Aging, University of Kentucky, Kentucky Alzheimer’s Disease Research Center, Lexington, KY 40536 USA; 5 Departments of Translational Neuroscience & Family Medicine, Michigan State University, Grand Rapids, and Michigan Alzheimer’s Disease Research Center, Ann Arbor, MI USA; 6 Department of Medical & Molecular Genetics, Indiana University School of Medicine, Indianapolis, IN USA; 7 Present Address: DePauw University, Greencastle, IN 46135 USA

**Keywords:** Psychiatric disorders, Predictive markers

## Abstract

Alzheimer’s disease (AD) is a progressive neurodegenerative disorder with loss of cognitive, executive, and other mental functions, and is the most common form of age-related dementia. Amyloid-β peptide (Aβ) contributes to the etiology and progression of the disease. Aβ is derived from the amyloid-β precursor protein (APP). Multiple microRNA (miRNA) species are also implicated in AD. We report that human hsa-miR20b-5p (miR-20b), produced from the MIR20B gene on Chromosome X, may play complex roles in AD pathogenesis, including Aβ regulation. Specifically, miR-20b-5p miRNA levels were altered in association with disease progression in three regions of the human brain: temporal neocortex, cerebellum, and posterior cingulate cortex. In cultured human neuronal cells, miR-20b-5p treatment interfered with calcium homeostasis, neurite outgrowth, and branchpoints. A single-nucleotide polymorphism (SNP) upstream of the *MIR20B* gene (rs13897515) associated with differences in levels of cerebrospinal fluid (CSF) Aβ_1-42_ and thickness of the entorhinal cortex. We located a miR-20b-5p binding site in the APP mRNA 3′-untranslated region (UTR), and treatment with miR-20b-5p reduced APP mRNA and protein levels. Network analysis of protein-protein interactions and gene coexpression revealed other important potential miR-20b-5p targets among AD-related proteins/genes. MiR-20b-5p, a miRNA that *downregulated* APP, was paradoxically associated with an *increased* risk for AD. However, miR-20b-5p also reduced, and the blockade of APP by siRNA likewise reduced calcium influx. As APP plays vital roles in neuronal health and does not exist solely to be the source of “pathogenic” Aβ, the molecular etiology of AD is likely to not just be a disease of “excess” but a disruption of delicate homeostasis.

## Introduction

Alzheimer’s disease (AD) is the most widespread age-related dementia and can overlap with other dementias, including vascular, Lewy body, and frontotemporal dementia, all of which currently have no cure, no effective treatment, and only minor palliative care [1]. AD affects millions worldwide and prevalence is expected to increase as the population ages. Understanding the disorder requires investigation of biochemistry, genetics, epigenetics, and classical neurobiology [2]. AD has no cure and limited long-term treatments. A renewed enthusiasm was kindled by aducanumab’s recent approval by the FDA as potentially the first disease-modifying anti-amyloid treatment [3], although this approval was accompanied by serious questions regarding the FDA’s decision and actual efficacy of the drug at reversing, halting, or even reducing cognitive decline [4]. Thus, the road remains open to research on biomarkers, small molecules that can penetrate the blood-brain barrier, and short noncoding RNA-based treatments Non-pharmacological strategies would open new avenues to tackle this devastating disease [5–8].

Pathologically, AD is characterized by the deposition of amyloid plaques [9] and tau tangles [10]. Plaque deposition is a consequence of the generation and aggregation of soluble amyloid β (Aβ) peptide. Once the Aβ peptide is generated, it can result in neuronal apoptosis via aberrant activation of plasma membrane expressed receptors, p75 neurotophin receptor [11, 12] and N-methyl-D-aspartate receptor (NMDAR) [13]. An increase in deposition and a reduction in clearance of this peptide may play a key role in the disorder [14]. Aβ peptide is cleaved out of a large transmembrane amyloid precursor protein (APP) by two enzymes sequentially, β-site APP-cleaving enzyme-1 (BACE1) and the γ-secretase complex [15, 16]. In addition, a major contributor to risk of AD is the presence of the *APOE*ε4 allele on the apolipoprotein E gene (*APOE*) [17–25]. This risk is sufficiently strong and widespread that we considered it necessary to examine potential *APOE*ε4 effects on outcomes of human tissue/subject based experiments, herein.

APP is a single-pass transmembrane protein found on both neurons and glial cells [15, 26, 27]. The protein is hypothesized to play roles in cell adhesion [28], neuroproliferation [29], neurite outgrowth [30], axonal guidance [31], and synaptogenesis [32]. Hence, regulation of APP levels is important to normal homeostatic function. APP knockout mice are viable but exhibit aberrant long-term potentiation (LTP), impaired locomotor activity, and reduced brain weight [33–35], thereby suggesting a necessary role in brain development, learning and behavior.

According to the amyloid cascade hypothesis [3], the elimination of amyloid plaque is expected to prevent or arrest AD progression, and, therefore, APP, BACE1 and γ-secretase have been selected as drug targets in the treatment of AD. Unfortunately, conventional drug therapies targeting these proteins have had limited success due to a variety of factors [3, 16, 36]. Thus, there is considerable focus on the identification of novel therapies to address the Aβ excess problem. MicroRNA (miRNA) manipulation is one such possible therapeutic strategy [37, 38].

MiRNAs are short, noncoding RNA that regulate expression of many proteins at the posttranscriptional level, primarily by targeting the mRNA 3′-untranslated region (UTR) [38]. Binding of miRNA to its cognate targets usually results in the reduction of protein expression either by inducing mRNA degradation or by inhibiting protein translation via interference with ribosome assembly. In the case of APP, miRNAs can target either the 3′-UTR or the 5′-UTR, subsequently modulating protein levels [39–43]. Levels of miRNAs also differ between post-mortem brains obtained from AD patients vs. non-cognitively impaired (NCI) controls. Therefore, miRNA targeting of amyloid metabolism may affect the onset and progression of AD and may constitute an important therapeutic strategy.

The focus of the present study is human hsa-miR20b-5p (miR-20b). Human miR-20b belongs to a cluster of the *MIR17* gene family that is highly conserved in vertebrates and negatively regulates APP expression [44–46]. The *MIR17* gene family consists of three paralogous groups located on chromosomes 7, 13 and X. Another member of the miR-17 family, miR-20a, is a negative regulator of APP expression [44–46]. This miRNA is 91% homologous to miR-20b-5p and their seed sequences are identical On the other hand, their precursor and mature sequences and their chromosomal locations differ [47].

In the present report, we identify miR-20b as a negative regulator of APP in human cell lines and a primary human brain (PHB) cell culture. We further show that elevated miR-20b associated with greater risk for AD in post-mortem brains. We also demonstrate that miR-20b’s reduction of APP expression is reversed by the addition of an antagomiR to miR-20b. Furthermore, we report that miR-20b can disrupt calcium homeostasis, neurite outgrowth and neuronal branchpoints in a primary human cell culture model. Finally, we identify a SNP approximately 15kb upstream of the *MIR20B* gene that is associated with AD-related cerebrospinal fluid (CSF) biomarker levels, specifically the 42 amino acid Aβ peptide (Aβ1-42) and with AD-associated neuroanatomical variation. These data take their place alongside our studies of miRNA regulation of BACE1, membrane metallo-endopeptidases (MME), and RE1 silencing transcription factor (REST) in PHB cultures and donated tissue from subjects who died with mild cognitive impairment (MCI) as well as AD and NCI subjects [48–53].

## Materials and methods

### Cell cultures

HeLa cells, human glioblastoma/astrocytoma (U373MG/U373), human microglia (HMC3), and human neuroblastoma (SK-N-SH) cells were procured from ATCC (American Type Culture Collection), and grown in EMEM (Corning) supplemented with 10% Fetal Bovine Serum (FBS) until they were ~70% confluent. After a brief trypsin digestion, cells were counted using the trypan-blue exclusion method. About 150,000 cells per well were seeded in a 24-well plate and left undisturbed overnight until transfection was carried out. For neuronal cultures, human neuroblastoma (NB) SK-N-SH cells were differentiated with 10 µM all-trans retinoic acid (ATRA, Sigma) for 7 days in 2% FBS maintenance media and afterwards referred to NBRA.

PHB cultures were grown as described previously [48]. Briefly, primary human embryonic brain tissues with no known gene mutations were obtained from the University of Washington Birth Defects Research Laboratory (Seattle), digested with trypsin, and plated in 24-well plates at a density of 150,000 cells per well. Media (neurobasal, Invitrogen) containing B-27 supplement at a ratio of 1:50, Glutamax (Invitrogen) at a ratio of 1:500, and basic fibroblast growth factor (bFGF, Invitrogen) was replenished every 4 days until 17 days in vitro (DIV 17). Transfections were performed from DIV 17 to DIV 20.

### APP 3′-UTR-coupled-reporter assay

The APP 3′-UTR was inserted downstream within a dual-reporter luciferase plasmid as described previously [41]. 50 nM of miR-20b or a commercially supplied negative control mimic (NCM) (ThermoFisher, lyophilized powder) were co-transfected with the full-length, 1.2 kb, APP 3′-UTR dual-reporter luciferase plasmid into HeLa cells and the effect of the miR-20b or NCM on luciferase reporter expression was assessed using the DualGlo luciferase assay (Promega) 48 h post-transfection.

### RNA transfection

Oligomer mimic of miR-20b (ThermoFisher) or a NCM was resuspended in nuclease free water and used at a concentration of 50 nM for HeLa experiments or 100 nM for the PHB tissue and human glioblastoma cultures. APP siRNA was used at a concentration of 20 nM for HeLa and 50 nM for PHB culture experiments. RNAiMax (ThermoFisher) was used as the transfection reagent at 1.5 µl/well. MiR-20b inhibitor or antagomiR (ThermoFisher) was used at a 100 nM dose in all experiments. Mock-transfected cells were treated with RNAiMax, but no miRNA. The transfection complexes were suspended in Opti-Mem media (ThermoFisher) and distributed 100 μl/well. The volume per well was brought up to 500 μl/well using the appropriate media. Cells were harvested at 72 h unless described otherwise.

### Cell harvesting

At 72 h post-transfection (or as indicated), conditioned media was collected and stored, and cells were washed with 1x PBS and lysed using 100 μL of Mammalian protein extraction reagent (M-Per, ThermoFisher) supplemented with one tablet of protease inhibitor cocktail (Roche). The cell lysate was centrifuged for 10 min at 15,000 × *g* and the supernatant was collected and used for Western blotting. Toxicity of experimental treatments was measured in cell cultures by lactose dehydrogenase (LDH) assay.

### RNA extraction and quantification from HeLa cells

For HeLa cultures both total RNA and proteins were extracted via the miRVana Paris kit (ThermoFisher) as per the manufacturer’s instructions and stored at −70 °C. RNA concentration and purity were measured using a Nanodrop spectrophotometer.

### RNA quantification by qRT-PCR

RNA from cell cultures was reverse transcribed with High Capacity RNA-to-cDNA kit (ThermoFisher). cDNA was subjected to real-time quantitative PCR (qPCR) analysis on QuantStudio 6 Flex instrument (ThermoFisher). Relative quantification was achieved by ΔΔCt (or “fold change”) normalization with the geometric means of housekeeping genes GAPDH and ACTB.

### Western blotting

An equal amount of protein from treatment samples was denatured by heating for 10 min at 95 °C in the Laemmle sample buffer. The denatured samples were loaded onto a 10% bis-tris, 26-lane gel (BioRad) and run at 200 V for 1.2 h. The gels were transferred onto PVDF membranes using the iBlot dry transfer system (ThermoFisher) and were blocked using 5% Milk in tris-buffered saline with Tween-20 (TBST). Primary antibodies used were as follows: anti-APP (clone 22C11; Millipore, 1:1000), anti-actin (Sigma, 1:500 000), anti-tubulin (Sigma, 1:500 000). After TBST wash, goat anti-mouse (1:3000) secondary antibody conjugated to horseradish-peroxidase was applied for 1 h. After washing, chemiluminescence “Super Signal” reagent (Pierce) was used to visualize protein bands.

### Human brain tissue specimens

We obtained well characterized autopsied tissue samples (Table 1) from cognitively normal older adults (non-cognitively impaired/NCI), subjects with MCI, or from subjects with AD. Three brain regions were examined, specifically: temporal lobe (TL, superior and middle temporal gyri, Brodmann areas 21/22) and cerebellum (CB, from lateral folia) from the University of Kentucky ADRC (P30 AGO072946), and posterior cingulate cortex (PCC, Brodmann areas 23/32) from the Rush University ADRC (P30 AG010161). We recently described these tissue resources in detail [49]. Briefly, samples were frozen at autopsy and stored at −80 °C. For experimental studies, samples were immersed in liquid nitrogen, powdered with a pestle, and subsequently lysed using M-Per buffer containing a protease inhibitor cocktail (Roche). After centrifugation at 13,000 × *g* for 15 min at 4 °C, supernatants (protein lysates) were heated in Laemmli sample buffer and separated on SDS-polyacrylamide electrophoresis (PAGE).

**Table 1:** Demographics of human brain samples.

Region^a^	Sex	Age (F, M)^b^
TL	12F, 10M	82.5 + 4.8/−2.8, 84.0 + 2.8/−6.8
CB	12F, 10M	82.5 + 4.8/−2.8, 84.0 + 2.8/−6.8
PCC	26F, 13M	89.6 + 1.9/−3.8, 85.9 + 3.5/−3.4

^a^TL: Temporal Lobe, CB: Cerebellum, PCC: Posterior Cingulate Cortex.

^b^Median age in years ± 75th/25th percentiles.

### RNA extraction from human brain tissue

RNA was extracted from frozen tissue using a modified Ambion PureLink mini kit protocol (#12183018A). Briefly, between 10 and 25 mg of tissue was placed in a 2 ml round bottom tube. One ml of Trizol (ThermoFisher #15596026) was added. Tissue was sonicated on ice until homogenous and then allowed to incubate for 5 min at room temperature. Then 200 μl of chloroform was added, and the sample was vortexed for 15 s. Following a 3 min incubation at room temperature, the samples were centrifuged at 12,000 × *g* for 15 min at 4 °C. The upper aqueous layer was transferred to a clean 1.5 ml tube, and an equal volume of 70% ethanol was added. The sample was vortexed and then processed following the manufacturer’s instructions. RNA was eluted in a final volume of 50 μl of nuclease free water, and then quantified to be used as a template for cDNA synthesis.

### MicroRNA quantification by qRT-PCR

Quantitation of miR-20b levels was determined using two methods, to wit: miR-20b levels in human tissue were analyzed by qPCR using both relative and absolute quantitative techniques. For relative quantitation, a probe-based assay for miR-20b (TaqMan 001014) was measured and compared to the control small RNA RNU48 (TaqMan 001006 labelled with VIC) [50]. Briefly, template for qPCR was generated using the TaqMan miRNA reverse transcription kit (ThermoFisher 4366596) following the manufacturer’s recommended protocol with an input of 10 ng of RNA. qPCR was performed on an ABI 7500 instrument in 20 μl reactions, which were incubated for 40 amplification cycles. Each reaction contained 1.3 μl of reverse transcription product as template, 2× master mix minus uracil-N-glycosylase (UNG) (ThermoFisher 444040), and each of the TaqMan assays listed above. Ct values were determined using a constant threshold, and fold change was calculated by the delta-delta Ct method.

For absolute quantification, the TaqMan Advanced cDNA synthesis kit (ThermoFisher A28007) was used to produce template for qPCR. Ten ng of RNA was polyadenylated, ligated to an adapter, reverse transcribed, and amplified, resulting in cDNA capable of being interrogated with any TaqMan Advanced miR assay. Then qPCR amplification reactions were assembled including 2 μl of miR-AMP product as template, 2× PrimeTime master mix (Integrated DNA Technologies 1055772), and TaqMan Advanced assay miR-20b (ThermoFisher 478430) in a total of 10 μl. The reactions were subjected to 40 rounds of amplification in an ABI 7500 thermocycler. A standard curve of not less than five data points was created using known concentrations of a miR-20b synthetic oligonucleotide (IDT, Coralville, IA). Ct values were determined using a constant threshold. Construction of the standard curve was performed by creating a scatter plot in Excel based on the Ct values of the samples of synthetic miR-20b. Concentrations of unknown samples were determined by extrapolation using the slope equation generated by the standard curve.

### Imaging and neurite measures

Transfected plates (mock or miR-20b treated) of PHB cells were placed in the Incucyte^®^ Zoom (Essen Bioscience) and imaged once a day to obtain four time points: 24, 48, 75, and-96 h. In a separate experiment, APP siRNA treated plate was imaged at 72 h only. Using the “neurotrack” analyzer, masking of cells and neurites were performed to ensure inclusion of cell bodies and neurites. Specifically, definition of minimum clusters was set to 50 µM^2^, segmentation adjustment was set to 1.2, minimum cell width was set to 5 µM, neurite filtering was set to ‘best’, neurite sensitivity was set to 0.35 and neurite width was set to 1 µM. These same parameters were used for all the conditions analyzed. The neurite lengths and branchpoints obtained were normalized to the clusters (an estimate of cell number within a field of view, using visual clusters as a proxy for cell bodies).

### Calcium imaging

PHB cells were grown on poly-D-lysine-coated coverslips (50,000 cells/well) in 35 mm dishes until DIV 17. Cells were transfected with either miR-20b or NCM for 72 h as described above. Ratiometric, calcium sensitive fluorescent dye Fura-2 (Catalog # F1221, ThermoFisher) was used to measure intracellular calcium level changes. Cells were loaded with Fura-2 by using its acetoxymethyl (AM) ester (Fura-2AM). On DIV 20, transfected cells were washed 3× PBS and loaded for 1 h with 4 μM Fura-2AM dye dissolved in 1 ml of HEPES buffered saline, followed by 3× PBS washes to remove unbound dye. Coverslips with Fura-2 loaded cells were kept in dark for 60 min to allow Fura-2AM to Fura-2 conversion before imaging was performed. Coverslips were placed on an inverted Zeiss microscope equipped with a back-illuminated Andor charge-coupled device camera. The coverslip was mounted into a perfusion system with continuous supply of either HEPES Buffer or HEPES buffer-containing 70 mM KCl. The coverslip was flushed with HEPES buffer for 10 min. During this time, regions of interest (ROI) were selected based on cell morphology; an attempt was made to include mostly non-aggregated cells. This was followed by a 5 min application of HEPES buffer containing 70 mM KCl to depolarize the neurons. Fura-2 was excited at 350 nm and 380 nm while the emitted light was collected using a 510 nm filter. The ratio of fluorescence intensities acquired at 350 nm (F350) and 380 nm (F380) excitation wavelengths was calculated for analysis. Only cells responding to KCl were selected for analysis, as they were likely neurons.

### Data for characterization of SNP in human tissues (ADNI)

Raw data used for analysis of *MIR20B* gene associated SNP effects were obtained from the Alzheimer’s Disease Neuroimaging Initiative (ADNI) database (adni.loni.usc.edu). These data included serial magnetic resonance imaging (MRI), positron emission tomography (PET), biological markers, and clinical and neuropsychological assessments (Table 2).

**Table 2:** Demographics of ADNI SNP samples.

Genotype	Number	Sex	Education^a^	Age^b^
A0	312	0 F 312 M	17 +2/−2	73.0 +5/−4
AA	229	229 F 0 M	16 +2/−3	72.0 +6/−6
GA	24	24 F 0 M	16 +2/−0.25	70.5 +3/−7.5
G0	14	0 F 14 M	18 +0/−3.5	71.5 +5/−2.25
GG	1	1 F 0 M	16 +0/−0	76.0 +0/−0

^a^Median years of formal education ± 75th/25th percentiles.

^b^Median years of age ± 75th/25th percentiles.

### Data analysis

Data were modeled for all experiments except brain level miR-20b analysis and entorhinal cortex thickness by generalized linear models (glm) followed by analysis of variance/deviance (ANOVA) and estimated marginal means using false discovery rate (fdr) to correct for multiple comparisons. A distinct advantage of the glm over ordinary least squares analysis is that the assumptions of normal distribution and similar variances (homoscedasticity) are greatly relaxed and do not need to be demonstrated for a valid analysis. Brain miRNA level data were analyzed by ordinal logistic regression (olr) followed by ANOVA. The olr presumes that the response variable is ordered, such as NCI, MCI, and AD representing progressive severity of condition. Since EC data were collected using two distinct MagFields, specifically 1.5TT and 3TT, we used a generalized mixed-level model with random intercepts for MagField to account for likely differences in measurements caused by the two settings. When MANOVA was used, assumptions of multivariate normality and sphericity were tested by Mardia’s and Bartlett’s tests, respectively. Data were found to be multivariate normal and to have homogeneous covariate matrices and was thus appropriate for MANOVA.

Sample sizes were determined by comparison to our earlier work, which has generated significant and repeatable results [40–42].

We calculated coefficients of determination (*D*) for ordinal logistic models via $D = mean\left( {p_c} \right) - \left( {mean\left( {p_c} \right) - 1} \right)$, where *mean*(*p*_*c*_) is the mean of each probability that corresponds to the specific level (in this case NCI/MCI/AD) reported in the data, and the second term is the remaining predicted probability sum for that point for input levels of the response variable is calculated. This method is an extension of Tjur’s *D* for binomial logistic models [55] and is analogous to the R^2^ coefficient of determination. To obtain values for each effect in the model, models were generated without an effect and *D* was calculated. Since *D* is an analogue for R^2^, the Fisher transformed Z of the full model and each sub-model were calculated from $\sqrt D$ subtracted from transformed $\sqrt D$ for the overall model. The result was back-transformed and squared to produce a partial *D* for each effect.

### Construction of interaction networks based on miR-20b and AD

We probed the STarMirDB utility with several AD-related mRNA sequences. Positive outcomes of this search were then used as inputs to NetworkAnalyst to map minimum networks for human hippocampus protein-protein interactions (PPI), frontal cortex PPI, hippocampus co-expression, frontal cortex co-expression, and signaling pathways. Proteins/genes found in these networks were then used as input to STarMirDB to investigate further miR-20b targeting. Networks were visualized by the R “igraph” package.

## Results

### miR-20b alters probability of AD, MCI, and NCI in elderly adult human brain samples

**Fig. 1:**
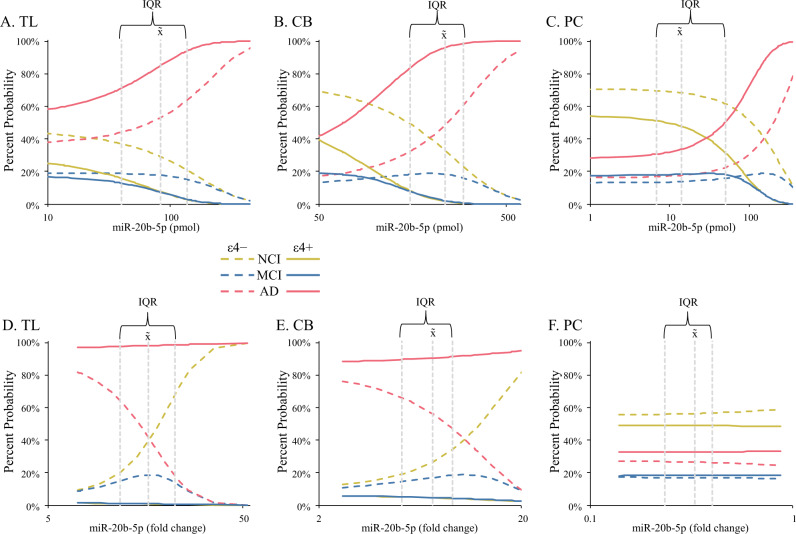
Levels of miR-20b by quantitative real-time PCR (qRT-PCR) in brain autopsy samples alter probability of NCI, MCI, and AD. miR-20b was quantified in temporal lobe (TL), cerebellum (CB), and posterior cingulate cortex (PCC) samples as described in the main text. Effect of miR-20b levels on probability of diagnosis (NCI/MCI/AD) was then modeled. **A–C** Comparison of absolute quantitation by diagnosis and brain region. **D**–**F** Comparison of ΔΔC_T_ (fold change) quantitation by diagnosis and brain region. Symbols indicate estimated marginal means categories. IQR: Interquartile range for miR-20b within a brain region. x̃: Median of miR-20b within a brain region. Significant effects are indicated by line colors and patterns, to wit: NCI, *APOE* ε4 absent: ; NCI, ε4 present: ; MCI, ε4 absent: ; MCI, ε4 present: ; AD, ε4 absent: ; AD, ε4 present:. Finally, miR-20b-5p axes for all charts is log scale.

Quantitation of miR-20b in *post-mortem* samples of NCI, MCI, and AD brains (Table 1) determined a complex relationship. Raw qRT-PCR signals were quantified by both absolute (Fig. 1A–C, Table 3) and fold change (Fig. 1D–F, Table 4) methods, then analyzed with ordered logistic regression to predict probabilities of each member of an ordered set of outcomes [56]. In this case, the order was NCI (no disease), MCI (mild/moderate disease), and AD (severe/worse disease).

**Table 3:** ANOVA of NCI/MCI/NCI vs. miR-20b levels (pmol).

Effect	χ^2^ (df)	*p*	*D*
miR-20b	13.48 (1)	<0.001	0.021
±*APOE*ε4	9.77 (1)	0.002	0.010
Region	3.57 (2)	0.168	0.004
miR-20b × ε4	2.08 (1)	0.149	<0.001

**Table 4:** ANOVA of NCI/MCI/NCI vs. miR-20b levels (fold change).

Effect	χ^2^ (df)	p	*D*
miR-20b	0.42 (1)	0.519	0.024
±*APOE*ε4	11.84 (1)	<0.001	0.098
Region	7.09 (2)	0.029	0.144
miR-20b × ε4	11.98 (1)	<0.001	0.039

Our model tested effects of miR-20b on diagnosis (AD/MCI/NCI) in different brain regions (TL, CB, PCC). We tested potential covariates of age, sex, and presence/absence of at least one *APOE* ε4 allele. For both absolute and relative quantitation, a model that included *APOE*ε4 presence/absence as a covariate was selected. When examining absolute quantities of miR-20b, differences by miR-20b levels and *APOE*ε4 presence had significant effects (Fig. 1A–C). *APOE* ε4 presence was associated with increased probability of AD overall, as expected. As miR-20b levels increased, probability of AD increased in all three brain regions. Relative quantitation of miR-20b produced a model where diagnosis had no independent association with miR-20b levels (Fig. 1D–F). However, *APOE*ε4 allele presence and diagnosis significantly interacted with regard to miR-20b levels. When the *APOE* ε4 allele was absent, miR20b levels associated with reduced probability of AD and increased probability of NCI. Effects were pronounced in TL and CB samples but were very weak in PCC. Comparing fit of models by *D* (Supplementary Table 1) showed that the relative miR-20b model had greater accuracy than the absolute quantified miR-20b model. Low levels of prediction for MCI may be an artifact of the sample, which consisted primarily of NCI and AD subjects.

### A single-nucleotide polymorphism (SNP) near miR-20b gene was associated with altered levels of CSF Aβ and entorhinal cortex thickness

**Fig. 2:**
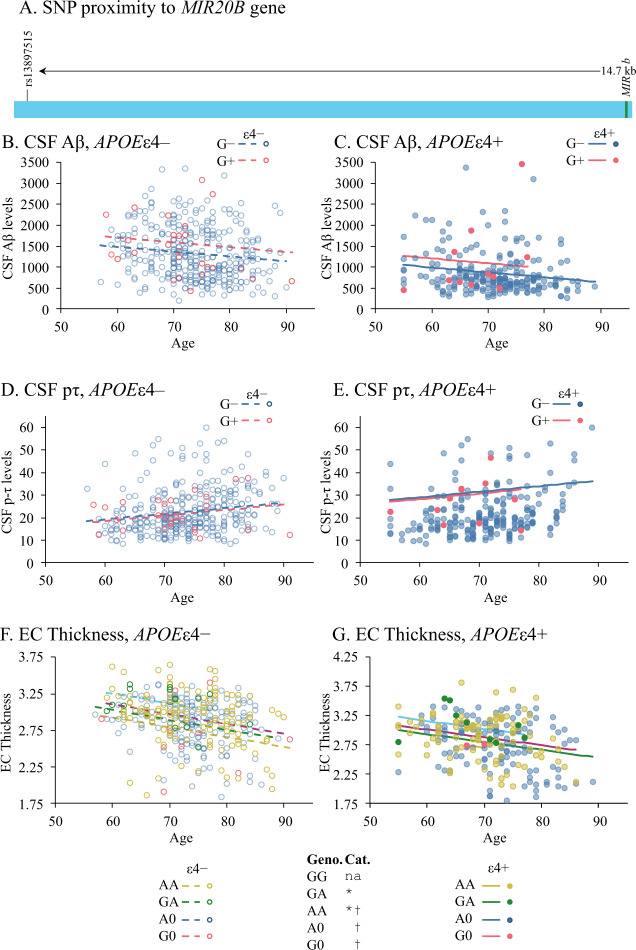
The rs138397515 SNP alters levels of CSF Aβ and thickness of entorhinal cortex. **A** Location of rs13897515 relative to *MIR20B* on X chromosome. **B**, **C** CSF Aβ levels were significantly influenced by G allele presence, age, and presence of the *APOE* ε4 allele. **D**, **E** CSF phosphor-tau (pτ) levels were significantly influenced by age and *APOE* ε4 allele presence but not by the SNP allele/genotype. **F**, **G** Thickness of the entorhinal cortex (EC) was significantly altered by SNP genotype, age and *APOE* ε4 allele presence. SNP and significant covariate effects are indicated by line/marker colors and patterns, to wit: G allele absent, *APOE* ε4 absent: ; G allele present, ε4 absent: ; G allele absent, ε4 present: ; G allele present, ε4 present: . For EC thickness, the most parsimonious model was based on genotype, with the corresponding representations: A0, ε4 absent: , G0, ε4 absent: ; AA, ε4 absent: ; GA, ε4 absent: ; A0, ε4 present: , G0, ε4 present: ; AA, ε4 present: ; GA, ε4 present: .

We queried the ADNI database for SNPs in or near the *MIR20B* gene on ChrXq26.2. One biomarker-linked SNP was identified (rs138397515). This SNP was 14.7 kb upstream of *MIR20B* and consisted of an A → G transition (Fig. 2A). Out of 580 subjects (Table 2), distribution among A0/AA/G0/GA/GG genotypes was 312/229/14/24/1, where “A0” and “G0” indicate male subjects. Hardy–Weinberg χ^2^ for X-linked traits determined that the alleles were within H-W equilibrium (*p* = 0.775). The “GG” genotype had only one representative and was excluded from further analysis. CSF biomarkers (CSF Aβ_1-42_ and CSF p-tau) from the dataset were compared to SNP variation (Fig. 2). Analysis was carried out on the 580 subjects as a single sample group. However, SNP effects were also tested vs. potential covariates of subject age, subject education (years of attainment), subject sex, and presence of at least one *APOE*ε4 allele. Higher CSF Aβ levels were associated with the presence of at least one G allele (Fig. 2B, C. *p* = 0.040, Table 5). CSF Aβ also was associated with subject age and presence of at least one *APOE*ε4 allele (*p* < 0.001). When we examined hippocampus volume and entorhinal cortex thickness, we found no association between rs138397515 status and hippocampus volume. However, the SNP genotype did associate with differences in entorhinal cortex thickness (Fig. 2F, G; *p* = 0.003, Table 5), specifically EC thickness increased along with genotype in the order G0 < A0 < AA < GA.

**Table 5:** ANOVA of SNP rs138397515 status effects.

Outcome	Effect	F (df, df)^a^	*p*	ω^2^
CSF Aβ	SNP G allele ±	1.38 (1, 577)	0.040	0.005
	Age	4.42 (1, 577)	<0.001	0.019
	*APOE*ε4 ±	32.11 (1, 577)	<0.001	0.148
CSF pτ	SNP G allele ±	0.23 (1, 577)	0.633	<0.001
	Age	14.69 (1, 577)	<0.001	0.021
	*APOE*ε4 ±	84.79 (1, 577)	<0.001	0.130
EC Thickness	Genotype^b^	14.04 (3)	0.003	0.012
	Age	36.39 (1)	<0.001	0.062
	*APOE*ε4 ±	4.48 (1)	0.003	0.015

^a^Statistic for EC thickness is χ^2^ (df).

^b^Genotypes in sample consist of A0, G0, AA, GA, and GG.

### miR-20b targets the APP mRNA through its 3’-UTR

**Fig. 3:**
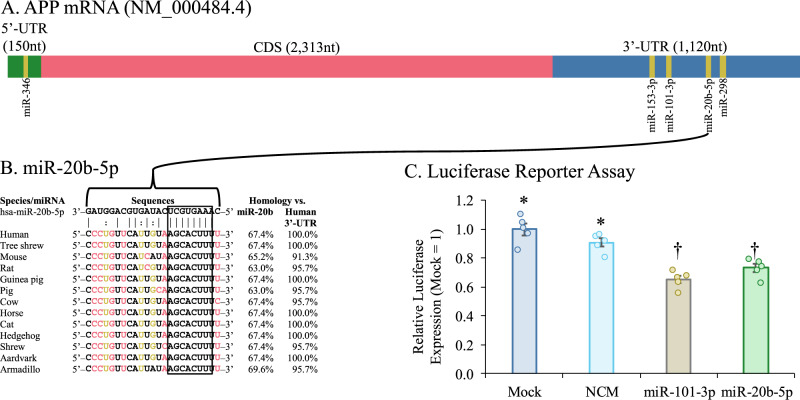
MiR-20b target conservation on APP 3′-UTR and site confirmation. **A** The hsa-miR-20b sequence was used to probe the APP 3′-UTR sequence with the STarMir utility. [57] A single putative site was predicted, with 100% homology to the 8-base seed sequence. Additional miRNA species previously confirmed to regulate APP translation are also shown [39–42]. **B** Multiple sequence alignment of homologous regions from 12 additional mammalian species. Seed sequence (outlined) was conserved across multiple genera and families. Homology was compared between each animal target sequence and hsa-miR-20b-5p sequence and vs. the human 3′-UTR target sequence. All sequences maintained at least 63% homology to complete miR-20b-5p sequence and at least 91.3% homology to the human target sequence. **C** APP 3′-UTR-coupled-reporter activity. Reporter clone with full APP 3′-UTR sequence was co-transfected with NCM and miR-101-3p and miR-20b mimics. miR-20b significantly (*p* ≤ 0.05) reduced levels of luciferase reporter expression. Symbols indicate estimated marginal means categories. Samples sharing a symbol did not significantly differ from each other. Error bars represent standard errors of means (SEM).

Query of the hsa-miR-20b sequence against the APP 3′-UTR via the StarMir algorithm [57] determined a putative site at base pairs 3158–3180 (Fig. 3A), predicted ΔG was −24.9. The site is highly conserved across multiple lineages of placental mammals, as shown by Multiz [58] alignment (Fig. 3B). We assessed the effect of miR-20b on the activity of the 3′-UTR in HeLa cells. We tested an *APP* 3′-UTR luciferase reporter clone [41] by treatment with mimic for miR-20b (Fig. 3C). Treatment reduced luciferase signal vs. mock and NCM treatment (*F* (df, df) = 24.136 (3, 16); *p* < 0.001; ω^2^ = 0.776). In addition, we transfected cells with miR-101-3p as a positive control. This oligo also reduced 3’-UTR reporter activity, confirming our previous results [41].

### Treatment by miR-20b alters levels of APP mRNA and protein

**Fig. 4:**
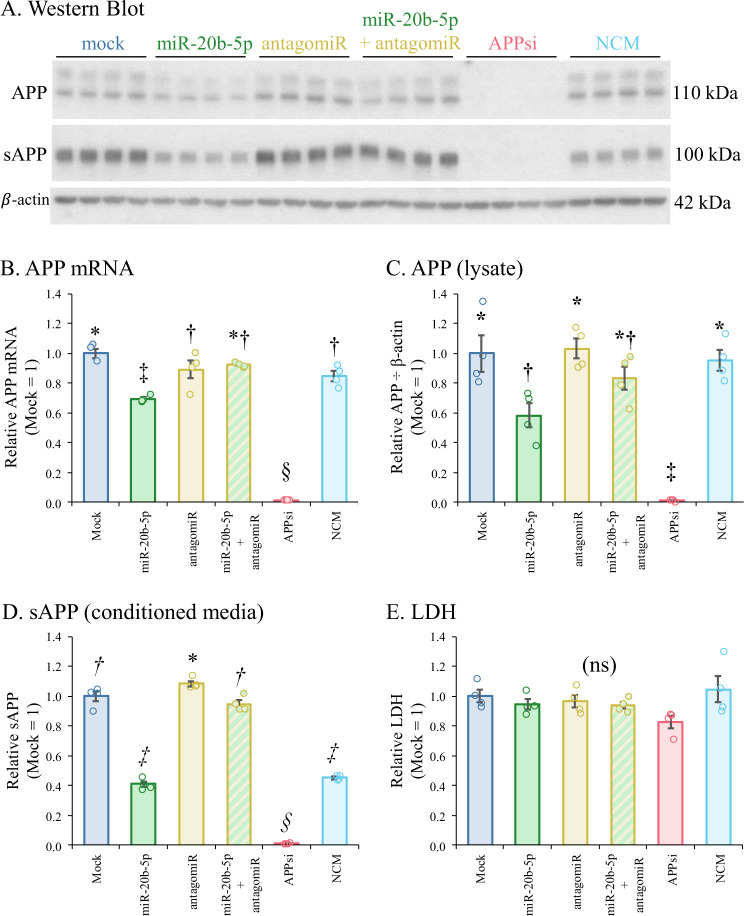
Treatment with miR-20b and related oligomers produces parallel outcomes for APP mRNA, intracellular APP, and secreted APP. HeLa cells were treated with oligomers: Specifically, miR-20b mimic, antagomiR to miR-20b, mimic + antagomiR, siRNA to APP, and a commercially obtained negative control mimic (NCM). Cultures and media were harvested and prepared for qRT-PCR, Western blotting, and LDH, as described in the text. Data were checked for multivariate normality then analyzed with MANOVA followed by estimated marginal means with the false discovery rate adjustment. Pillai’s trace (df, df): 2.17 (5, 1); *p* < 0.001, η^2^ = 0.542. **A** Western blot of intracellular and secreted APP (sAPP), along with β-actin. **B** qRT-PCR relative outcomes, mock = 1. **C** Relative densitometry of intracellular APP, mock = 1. **D** Relative densitometry of sAPP, mock = 1. **E** Relative LDH signal, mock = 1. Symbols indicate statistical categories. Samples sharing a symbol do not differ at *p* ≤ 0.05. Error bars represent standard errors of means (SEM).

Treatment of HeLa cells by miR- 20b mimic significantly reduced levels of APP mRNA (Fig. 4B), intracellular APP (Fig. 4A, C), and secreted APP (sAPP) in conditioned media (Fig. 4D). Treatment by siRNA against APP resulted in even greater reduction of these outcome measures. Co-treatment with miR-20b and an antagomiR against miR-20b prevented and reversed this response, respectively. Cell culture toxicity, as measured by LDH release, was not altered by any treatments (Fig. 4E). Of note, the NCM treatment significantly reduced sAPP levels. However, since this was not accompanied by reduction in APP mRNA or intracellular APP protein, this outcome may be a result of an interaction between the NCM oligomer and APP cleavage or secretion protein mRNAs. As a multivariate outcome experiment, this was analyzed by MANOVA after confirming multivariate normality by Mardia’s test and sphericity by Bartlett’s test. (Pillai’s trace (df, df): 2.17 (5, 1); *p* < 0.001, η^2^ = 0.542).

### miR-20b reduced levels of APP in other human cell cultures

**Fig. 5:**
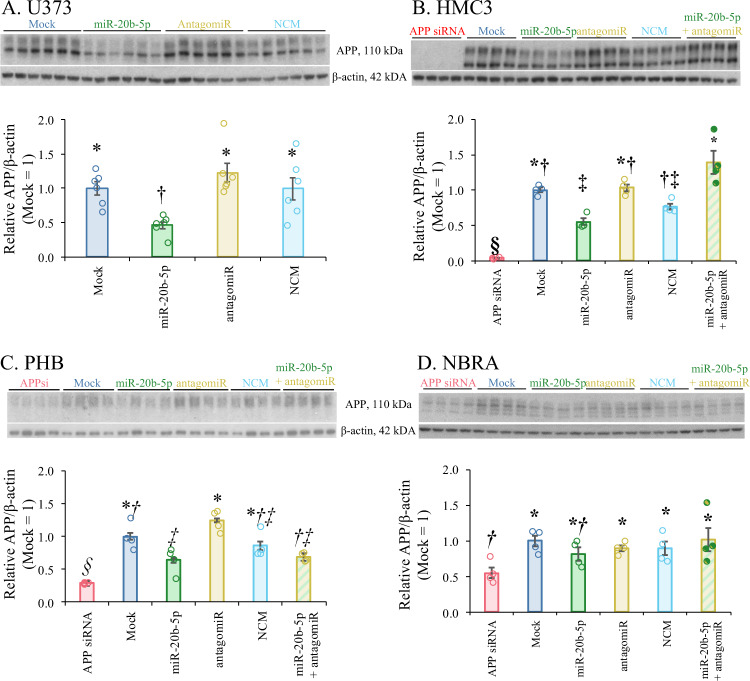
Treatment with miR-20b reduced levels of APP in U373 astroglial and HeLa epithelial cells, while miR-20b reduced levels of APP in HMC3 microglial cells and in human primary brain cultures (PHB) and was reversed by co-treatment with antagomiRs. U373, HMC3, PHB, and NBRA cells were cultured and treated with miR-20b mimic or antagomiR as specified in the main text. **A** Western blot and densitometry of APP and β-actin in U373 cells. miR-20b significantly reduced levels of APP (relative to β-actin). **B** Western blot and densitometry of APP and β-actin in HMC3 cells. Like U373, miR-20b significantly reduced levels of APP. **C** Western blot and densitometry of APP and β-actin in PHB cultures. miR-20b reduced levels of APP. **D** Western blot and densitometry of APP and β-actin in NBRA cells. miR-20b did not reduce levels of APP measured. Co-treatment with miR-20b antagomiR eliminated miR-20b reduction of APP in U373, HMC3, and PHB. Error bars represent standard errors of means (SEM).

We further transfected miR-20b in several additional human cell cultures, including astrocytic (U373), microglial (HMC3), differentiated neuroblastoma (NBRA), and PHB cultures. To explicitly test whether apparent differences in response among cell lines could be detected, we tested a model of APP ~ Treatment + Line + Treatment × Line, each blot normalized to “Mock = 1”. We discovered that cell line, treatment, and the interaction of treatment and cell line/culture were significant (Table 6), indicating that apparent response differences among cell lines/cultures were also significant (Fig. 5). Treatment of miR-20b mimic reduced APP levels in U373 cells (Fig. 5A). MiR-20b treatment also significantly reduced levels of APP in HMC3 (Fig. 5B) and PHB cultures (Fig. 5C). When ATRA-differentiated neuroblastoma cultures (NBRA) were treated with miR-20b mimic, APP reduction was not significant (Fig. 5D).

**Table 6:** ANOVA of APP levels vs. oligomer treatments in multiple cell lines.

Effect	F (df, df)	p	D
Treatment	6.39 (6, 90)	<0.001	0.188
Cell Line	0.32 (7, 90)	0.926	<0.001
Treatment × Line	3.15 (20, 90)	<0.001	0.214

### miR-20b and APP siRNA treatments each reduce calcium influx in a developmental human brain model culture

**Fig. 6:**
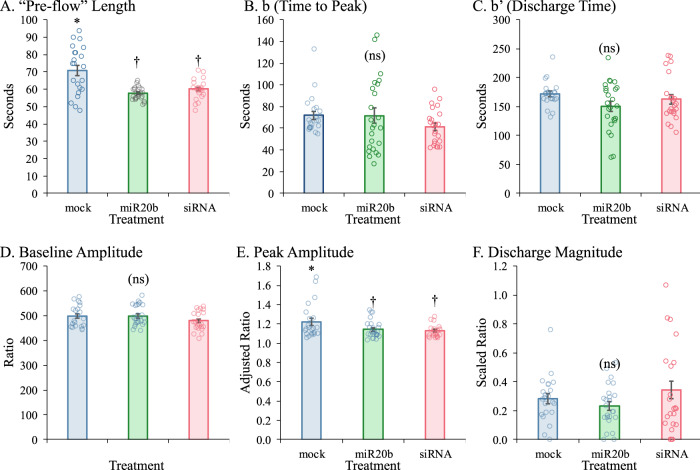
Effects of miR-20b treatment on calcium efflux. Outcomes for different measures were calculated as described in main text. **A** “Pre-flow” length, indicating time between injection and first response. **B** Time to peak (cycles) starting at point “0”. **C** Discharge time (between peak and “II”). **D** Baseline (“0” to “I”) amplitude. **E** Peak amplitude. **F** Discharge magnitude. Symbols indicate statistical groups of response over entire curve. Treatments sharing the same symbol did not significantly differ. Error bars represent standard errors of means (SEM). Lack of any pairwise significant differences indicated by “(ns)”.

We tested the effect of miR-20b and APP siRNA on calcium influx of PHB using Fura-2 imaging (Fig. 6, Table 7). An “idealized” Fura curve (Supplementary Fig. S1A), would progress through “pre-flow”, from first cycle “0” to “I”, which is the beginning of the influx. Influx would then progress to “P”, the peak. It would then be followed by active discharge to “II”, after which it would be passive tailing off. The distance from I to P, “b”, is the “time to peak”. The distance “b’” from P to II is the “discharge time”. The height “a” is the “peak amplitude”, and the height “a’” is the “ratio decay magnitude”. Six comparisons were made based on different normalizations of the Fura-2 curves. Length of “pre-flow” and heights of 350/380 signal for this segment were based on raw data (Supplementary Fig. S1B). The length of “pre flow” was significantly longer for mock than for miR-20b or siRNA treated cells (Fig. 6A). However, 350/380 ratios did not significantly differ among treatments (Fig. 6B). To compare distances b, b’, and a (peak height), positions of I for each treatment were normalized and trace heights were normalized to one baseline. Neither distances b (Fig. 6C) or b’ (Fig. 6D) significantly differed among treatments. However, mean peak amplitude (Fig. 6E) was significantly higher for mock-treated cells than for either miR-20b or siRNA treated cells. However, ω^2^ (less-biased analog of η^2^) was quite small (0.093). To compare distance a’ (discharge magnitude), traces were scaled to minimum and maximum of each trace (min = 0, max = 1). No significant differences were found by treatment (Fig. 6F). In sum, while total influx was reduced by both miR-20b and APP siRNA, other elements of the process were not altered.

**Table 7:** ANOVA of Fura experiment outcomes.

Outcome^a^	*F* (df, df)	*p*	ω^2^
“Pre-flow” Length	16.73 (2, 66)	<0.001	0.314
b (Time to Peak)	1.41 (2, 66)	0.253	0.012
b’ (Discharge Time)	1.93 (2, 66)	0.154	0.026
Baseline Amplitude	2.33 (2, 66)	0.104	0.037
Peak Amplitude	4.53 (2, 66)	0.014	0.093
Discharge Magnitude	1.25 (2, 66)	0.294	0.007

^a^Outcomes are measured and analyzed separately.

### miR-20b modulates neurite outgrowth and branchpoints in a developmental human brain model culture

**Fig. 7:**
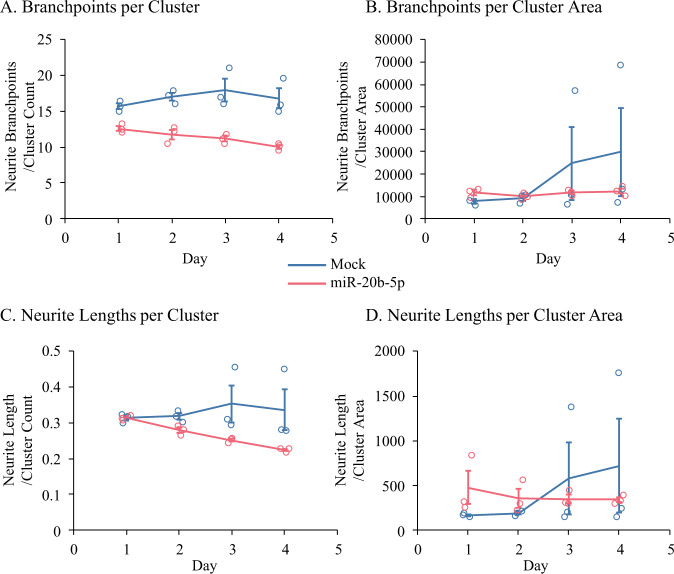
Treatment with miR-20b reduced neurite branchpoints in PHB cultures. Cells were subjected to Mock or miR-20b treatment as indicated. Neurite branchpoints were adjusted by (**A**) Cluster count or (**B**) Cluster area. Neurite lengths were adjusted by (**C**) Cluster count or (**D**) Cluster area. For both neurite branchpoints and neurite lengths, treatment with miR-20b resulted in reduced neurite branchpoints and neurite lengths, regardless of adjustment, over time vs. Mock treatment. Error bars represent standard errors of means (SEM) at each time point.

We tested whether transfection of miR-20b modulates neuronal morphology in our PHB cultures. Both neurite branchpoints (Fig. 7A, B, Table 8) and neurite lengths (Fig. 7C, D, Table 8) were reduced over time (*p* < 0.001) when compared to mock-transfected cells, following miR-20b treatment.

**Table 8:** ANOVAs of neurite morphological outcomes.

Outcome^a^	Effect	*χ*^2^ (df)	*p*	ω^2^
Branchpoints per Cluster	Treatment	29.59 (1)	<0.001	0.603
	Day	10.34 (1)	0.001	0.027
	Treatment × Day	25.07 (1)	<0.001	0.166
Branchpoints per Cluster Area	Treatment	2.93 (1)	0.087	0.043
	Day	41.08 (1)	<0.001	0.388
	Treatment × Day	34.22 (1)	<0.001	0.282
Neurite Lengths per Cluster	Treatment	5.87 (1)	0.015	0.111
	Day	16.24 (1)	<0.001	0.053
	Treatment × Day	33.12 (1)	<0.001	0.427
Neurite Lengths per Cluster Area	Treatment	17.29 (1)	<0.001	0.158
	Day	22.33 (1)	<0.001	0.533
	Treatment × Day	31.77 (1)	<0.001	0.145

^a^Outcomes are measured and analyzed separately.

### Network analysis revealed multiple potential miR-20b targets that also interact with AD-related proteins/genes

**Fig. 8:**
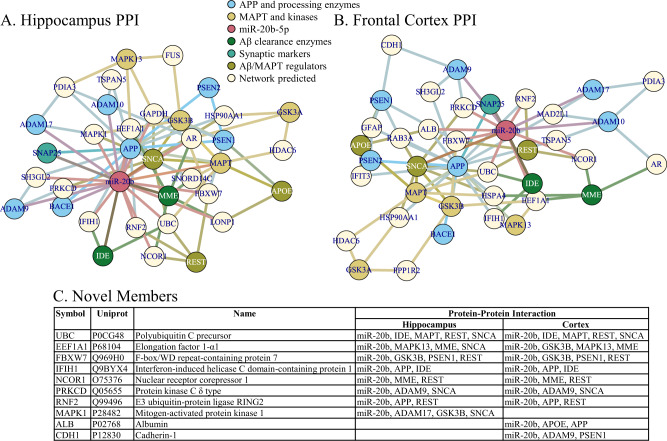
Protein-protein interaction networks of miR-20b, AD-related targets. Networks were generated by NetworkAnalyst as described in the text. Network nodes are color coded by protein function related to AD. **A** Hippocampus PPI network. **B** Frontal cortex PPI network. **C** Summary of network members in addition to “seed” members (or “novel” members).

**Fig. 9:**
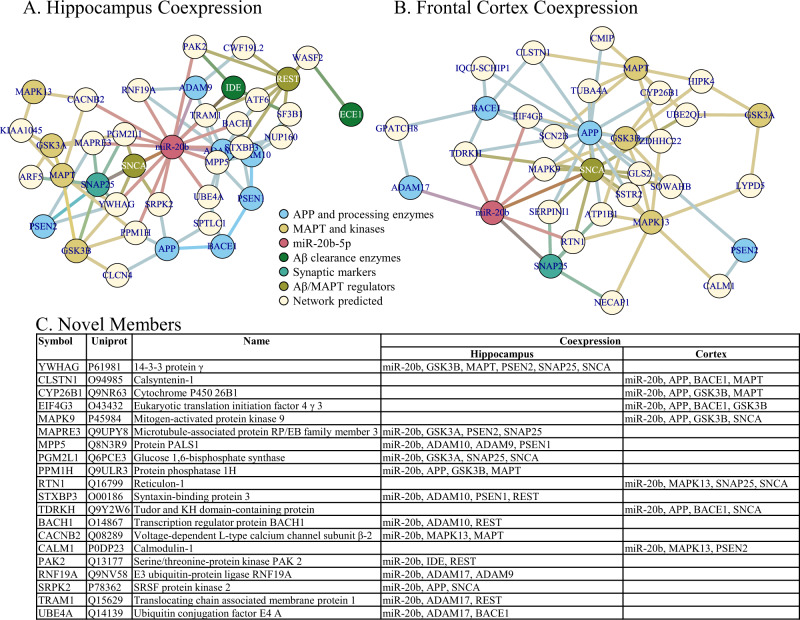
Coexpression networks of miR-20b, AD-related targets. Networks were generated by NetworkAnalyst as described in the text. Network nodes are color coded by protein function related to AD. **A** Hippocampus PPI network. **B** Frontal cortex PPI network. **C** Summary of network members in addition to “seed” members (or “novel” members).

**Fig. 10:**
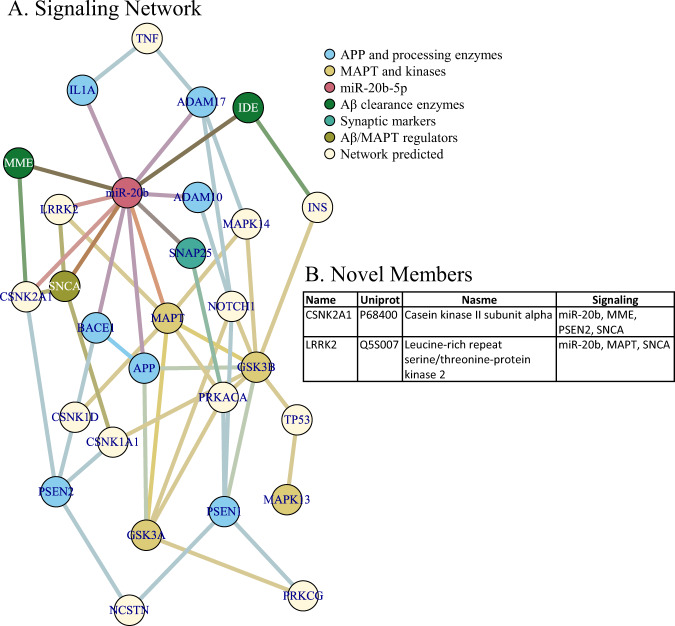
Signaling network of miR-20b, AD-related targets. Network was generated by NetworkAnalyst as described in the text. Network nodes are color coded by protein function related to AD. **A** Signaling network. **B** Summary of network members in addition to “seed” members (or “novel” members).

When we constructed networks based on miR-20b targeting of AD-related mRNA sequences, we first noted that miR-20b had predicted binding sites in the mRNA sequences of multiple AD-associated gene products (Table 9). We generated five networks (Figs. 8–10), specifically Protein-Protein Interaction (PPI) for human hippocampus (Fig. 8A) and human frontal cortex (Fig. 8B); co-expression for human hippocampus (Fig. 9A) and human frontal cortex (Fig. 9B); and signaling pathways (Fig. 10). Upon closer examination, we found multiple hits in each network that were (1) not in the original probe list, (2) interacted with more than one AD-associated proteins, and (3) predicted to be targeted by miR-20b. What may be interesting is that none of the network types (PPI, coexpression, and signaling) overlapped for any of these discovered network members, even within the same brain region. On the other hand, within network type, there was extensive overlap for PPI between hippocampus and frontal cortex but no overlap between hippocampus and frontal cortex for coexpression. Literature search reveals that the majority of these proteins may have some function in AD, and expanding analysis of miR-20b to include these targets in AD research may be fruitful.

**Table 9:** Major AD-related proteins used as network building seeds.

Protein	Uniport	Full name	Category^a^	miR-20b^b^
ADAM10	O14672	Disintegrin and metalloproteinase domain-containing protein 10	Amyloid	+
ADAM17	P78536	Disintegrin and metalloproteinase domain-containing protein 17	Amyloid	+
ADAM9	Q13443	Disintegrin and metalloproteinase domain-containing protein 9	Amyloid	+
APOE	P02649	Apolipoprotein E	Regulator	
APP	P05067	Amyloid precursor protein	Amyloid	+
BACE1	P56817	Beta-secretase 1	Amyloid	+
ECE1	P42892	Endothelin-converting enzyme-1	Clearance	
GSK3A	P49840	Glycogen synthase kinase-3 alpha	Tau	
GSK3B	P49841	Glycogen synthase kinase-3 beta	Tau	
IDE	P14735	Insulin-degrading enzyme	Clearance	+
IL1A	P01583	Interleukin-1 alpha	Amyloid	+
IRP1	P21399	Iron-responsive element-binding protein 1	Amyloid	
IRP2	P48200	Iron-responsive element-binding protein 2	Amyloid	
MAPK13	O15264	Mitogen-activated protein kinase 13	Tau	
MAPT	P10636	Microtubule-associated protein tau	Tau	+
MME	P08473	Membrane metallo-endopeptidase	Clearance	+
PSD95	P78352	Postsynaptic density protein 95	Synaptic	
PSEN1	P49768	Presenilin-1	Amyloid	
PSEN2	P49810	Presenilin-2	Amyloid	
REST	Q13127	RE1-silencing transcription factor	Regulator	
SNAP25	P60880	Synaptosomal-associated protein 25	Synaptic	+
SNCA	P37840	Alpha-synuclein	Regulator	+
SYPH	P08247	Synaptophysin	Synaptic	

^a^Classification vs. AD relationship, specifically, Amyloid: APP, APP processing enzyme, or APP translation factor; Clearance: Aβ clearing enzyme; Regulator: Protein with functions/effects on both Aβ and hyperphosphorylated tau protein; Tau: either MAPT or one of its major kinases.

^b^Predicted or confirmed to interact with miR-20b-5p.

## Discussion

Our integrated studies used different approaches, such as bioinformatics, biochemical, cellular, genetics, physiology and neuropathology, to reveal potential novel roles for miR-20b in AD. We showed that miR-20b represses levels of APP by targeting a predicted 7-mer m8 on the APP 3′-UTR. This repression was seen in epithelial, astrocytic, microglial, and a primary human mixed-brain cell culture model. This work adds to other reports of miRNA regulating APP levels [39–42, 44, 54, 59, 60]. Our cell culture work also showed that miR-20b reduces APP 3′-UTR activity and protein. Furthermore, we demonstrated that miR-20b and APP siRNA reduce intracellular Ca^2+^ transients in response to neuron membrane depolarization. Since our work also shows that miR-20b-5p reduces APP in microglial cells, future work should address whether the miRNA can also affect intracellular calcium levels in microglial cells. Treatment by miR-20b also resulted in reduction in neurite branchpoints and neurite length in PHB cell cultures. A SNP near *MIR20B* gene was associated with significant differences in a CSF biomarker (CSF Aβ_1-42_) levels and thickness of the entorhinal cortex detected via MRI. Finally, AD progression (NCI/MCI/AD) corresponded to increases in brain miR-20b levels when miRNA was measured by pMol, and we found evidence of interaction of *APOE* ε4 allele presence and miR-20b levels.

Our work adds knowledge about miR-20b’s important role in vital cellular- and organ-level pathways. For instance, miR-20b negatively modulates many different targets including endothelial PAS domain 1 [61], vascular endothelial growth factor in the lung [62], ephrin B2 and B4 in human placental tissue [63], proteinase-activated receptor-1 [64], phosphatase and tensin homolog [65, 66], signal transducer and activator of transcription 3 [67], induced myeloid leukemia cell differentiation protein Mcl-1 [68], B-cell translocation gene [69], IL-1 receptor-associated kinase 4 [70], hypoxia-inducible factor 1-alpha [71], and protein kinase B [72].

We report that transfection of miR-20b reduced both synaptic branch lengths and branchpoints in human neuronal culture. APP is expressed in the presynaptic membrane and is trafficked along via vesicles in the pre-synapse [73, 74]. Similarly, Aβ fragments are generated at both the pre-synaptic and post-synaptic neurons [75]. Previous research suggests that dimerization of APP is important to neurite growth [76], and, therefore, it is possible that our observed miR-20b effect on neurite outgrowth influences APP dimerization. It may be noteworthy that, in addition to APP and Aβ synaptic activity, BACE1 also exerts control on synaptic function [77]. The neurite-based measures of cells treated with miR-20b experienced a noteworthy increase in variability on days 3 and 4. This was most pronounced when branchpoints and length were normalized to cell body area. Such variation could be technical or biological. Technically, culture cell confluence was high, which was necessary to maintain health of primary human brain cultures. However, such density may have interfered with accurate determination of cell body number (clusters) and areas, as well as with visibility of branch points and neurites. Multiple layers may have obscured accurate measurements. In addition, cell debris from transfection may have been misidentified. However, given that transfection was a single early event, cell debris would have remained constant or decreased. Biologically, cell count and body area variation very well may have increased over time. Representative photomicrographs (Supplemental Figure S2) indicate a decrease in apparent cell density (by visual examination). Confirming biological explanation might require single-cell analysis for effects of miR-20b treatment, wherein each cell may have received a differing “micro-dose” or each cell had a slightly different response to miR-20b.

Loss of synaptic integrity and function is a component of the pathologic loss of neuronal function seen in AD [78]. Maintenance of synaptic architecture is crucial to the overall brain function. Calcium-dependent vesicular-mediated neurotransmitter release is dependent on the activation of voltage-gated calcium channels in response to a depolarizing action potential traveling down the axon. However, the presence of high calcium in the cell can result in neuronal death due to excitotoxicity. The cell attempts to stabilize internal calcium levels by sequestering calcium into intracellular compartments such as the sarcoplasmic reticulum. Therefore, the cytosol and the presynaptic membrane have a low calcium concentration under normal physiological conditions.

An APP overexpression experiment could conclusively demonstrate that miR-20b alters calcium influx and neurite outgrowth via an APP-mediated pathway. However, multiple studies have already reported that APP promotes neurite outgrowth both in vitro and in vivo. For example, APP increases axonal arborization in Drosophila brains [79]. APP knockdown by shRNA inhibits neurite outgrowth in neuron cultures, while APP knockout in vivo reduces neurite numbers and lengths [80]. Similarly, APP regulates intracellular calcium levels. Expression of human APP in rat cortical neurons increased calcium influx [81]. Based on our data, APP siRNA and miR-20b treatment both reduce calcium influx. We can infer that miR-20b reduces calcium influx at least in part due to APP downregulation. Nevertheless, we still must admit that we do not know whether miR-20b alters expression of other proteins independent of APP, which in turn regulate neurite outgrowth and ion channels. Future work should involve a comprehensive “-omic”-type experiment.

Small changes in the membrane expression of calcium-permeable channels can result in large-scale cellular events. These can be beneficial, for example, in the insertion of NMDAR receptors during the events of LTP. Aβ is involved in LTP and normal memory processes [82–85], suggesting that excess removal of Aβ may impact learning. APP may itself modulate trafficking of NMDAR to the membrane [86]. Therefore, identifying miRNA that may influence Aβ levels would be beneficial to our understanding of Aβ’s role in the synapse.

**Fig. 11:**
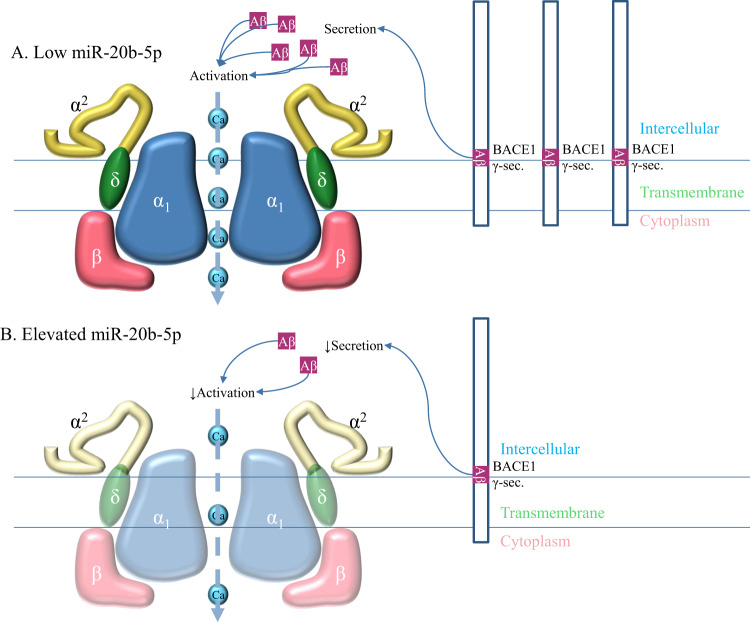
Potential mechanisms of miR-20b dependent effects on calcium channel activity. **A** Under conditions of low miR-20b, subunits of VGCC are produced at higher levels, along with APP. APP is processed by BACE1 and γ-secretase complex to produce Aβ, which promotes VGCC activity, maximizing Ca influx, possibly by lowering the channel’s activation threshold [87]. **B** Under elevated miR-20b, protein translation of multiple VGCC subunits may be (partially) inhibited, reducing VGCC levels in the cell membrane. Furthermore, downregulation of APP results in reduced levels of Aβ, which decreases VGCC activity. Overall effect is reduced movement of Ca across cell membrane. Different domains of APP (right panel) are as shown: Intercellular, Transmembrane and Cytoplasmic.

Taken together, our data suggest that increasing miR-20b levels results in loss of neuronal function, as measured by neurite length and branching, as well as a reduction in KCl-evoked intracellular calcium influx. We posit two potential mechanisms (Fig. 11). First, miR-20b reduces voltage-gated Ca^2+^ channels (VGCC) expression directly. A search of several neuronal subunits of VGCC revealed multiple potential miR-20b binding sites (Supplementary Tables 2–3). Nevertheless, we speculate that the reduction observed may also be at least in part due to miR-20b reversing calcium influx by reducing APP. This may induce a subsequent reduction of available Aβ to insert into the membrane and potentially to regulate VGCC function, possibly by lowering the channel’s activation threshold [87], specifically because our APP siRNA transfection induced a response that was similar to that induced by miR-20b.

We successfully measured miR-20b in post-mortem brains of NCI, MCI, and AD patients. This adds to previous work that measured miR-20b in circulating CNS-derived exosomes in the blood [88–90] as well as in peripheral tissue [91, 92]. Our work suggests levels of miR-20b differ in post-mortem brains of AD vs. NCI patients, when miR-20b is absolutely quantified. By contrast, using the ΔΔC_T_ (fold change) method resulted in no detected differences. In addition, our analysis suggests that miR-20b may increase in older individuals suffering from AD, thereby suggesting a novel interaction of changing miRNA levels with age. This observation should be investigated further. It is also important to note that our brain samples were limited to three brain regions: the PCC, the cerebellar cortex, and the TL. Therefore, future studies should measure levels of miR-20b in other brain regions, such as hippocampus and entorhinal cortex.

Our data suggest that the rs138397515 (A/G) SNP, located upstream of the *MIR20B* gene on Chromosome X, is associated with increase in CSF Aβ_1-42_. In addition, the SNP status was associated with differences in entorhinal cortex thickness detected by MRI. According to the ALFA project [93], studying the genomes of ALzheimer’s patients and their families, frequency of the minority “G” allele varies little among many populations (0.02–0.03) except among Asian populations, where the allele is not recorded and African-origin populations, where frequency is ~0.003 [93]. How miR-20b expression is affected by this upstream SNP will require further investigation. This SNP has the potential to participate on the complexity of effects influencing AD onset in disparate racial and ethnic groups. While apparent racial differences in AD have been reported [94], it remains unknown to what extent these differences have genetic, environmental, iatrogenic, or other unknown basis [95].

An apparent paradox underlies our present work. We demonstrated by cell culture work that application of miR-20b reduces levels of APP. However, miR-20b appears to be increased in one model relating miR-20b levels to probability of AD. Likewise, elevated levels of miR-20b appear to hamper neuronal communication (measured by neurite growth) and calcium response. Notably, the model that represented miR-20b in brain by relative quantification (−ΔΔC_T_/fold change) showed a definite *decrease* in probability of AD as miR-20b increased, and this model has a higher *D* (R^2^ analogue) than did the model based on absolute quantification of miR-20b. In addition, while APP and Aβ aggregation are associated with AD pathogenesis and pathology, APP serves necessary functions, these functions may differ in developmental vs. late-life stages. For example, APP plays a vital role in neuronal pruning [96], ensuring normal development of the brain. Insufficient pruning is widely accepted as a potential cause of autism spectrum disorder (ASD) [97, 98]. Here we used PHB culture as a proxy of a “developmental model”.

Taking the different outcomes and models used into account, we propose that one function of miR-20b in APP regulation is to act as a part of pruning mechanisms. APP is an adhesion protein that facilitates neurite outgrowth and proliferation at developmental stages. Suppressing such activity in a developmental model (i.e., PHB) would result in reduction in neurites and their lengths. Likewise, calcium signaling plays a vital role in neurodevelopment. The suppression of signaling by reducing APP may be a component of the pruning process (Supplementary Fig. S3). At the other end of life, in the aged individual, miR-20b downregulation of APP would help maintain lower Aβ levels, reducing excitotoxicity, aiding neuropreservation. However, if APP is de-repressed, neurodegeneration could ensue from reversing the process of blocking Aβ production (Supplementary Fig. S3C, D). Finally, our exploratory network analysis generated multiple potential novel targets connecting miR-20b regulatory activity with AD-related pathways and mechanisms. Future studies will address these pathways as well as functional interactions between miR-20b, APP, and neurodegenerative processes including Ca^2+^ dyshomeostasis and synaptic loss.

In this context, we have noted several potential links between AD and other disorders, such as ASD, primarily through APP [98–101]. The present work further connects AD and ASD through APP and its metabolites and regulation, specifically in the realm of neuron growth and development.

We would be remiss were we not to provide a cautionary statement. Specifically, several conclusions are based on the use of PHB cultures. We used these cultures because they offer advantages over animal-based primary cultures (since PHB cultures are human) and over induced pluripotent stem cell cultures (iPSC) (since the PHB have not been as manipulated). In addition, they are, perforce, a mixed-cell type culture that is a better representation of human brain than is any pure cell type culture. However, this also means that the specific output of each cell type remains unknown, which reflects actual human brains, wherein each “pure” type of cell nevertheless is exposed, at greater or lesser levels, to other types of cells within the brain. Furthermore, PHB cultures are not genetically homogeneous but each is unique genetically and by age (of harvest). This also turns out to better resemble the reality of human populations than does a genetic monoculture. On the other hand, obtaining the stocks for human fetal brain cultures is more challenging than for traditional immortalized cell cultures or for iPSC, making it difficult to obtain sufficient power for all but the most clear-cut experimental results.

In future work, we would consider exploring larger numbers of samples, with broader age ranges (as possible), and different sources of donation, including the possibility of culturing mature brain samples [102] obtained from volunteers undergoing brain surgery. In addition, our Fura data, however apparently clear-cut, require independent validation. Our mechanistic experiments in cell lines, PHB culture, and patient autopsy brains, would benefit from extension into mouse models [103–105], which would also permit testing organism effects of miR-20b modulation. We avoided mixing human and mouse systems in the present work due to potential for confounding species divergence of miRNA activity [106, 107]. This would be addressed by potentially using the mouse miR-20b or ensuring that AD-related transgene inserts included that relevant human miR-20b recognition sequences, including human 5′ and 3′ UTRs. We recognize that it would be tempting to do animal work in a miR-20b overexpression model, since this could potentially overcome difficulties of exogenous miR-20b administration (transient transfection). However, it would hardly be a valid model for potential treatment of AD, given that transgenic induction of miR-20b overexpression in humans would not be a practical treatment. In addition, since the familial AD types that underlie most mouse models only represent minority of human AD cases, we would also investigate inducible mouse AD models [108].

Furthermore, three clinical outcome instruments (Alzheimer’s Disease Assessment Scale–Cognitive Subscale (ADAS-COG), specifically ADAS-COG 13-item [109], Mini-Mental State Examination [110], and Rey Auditory Verbal Learning Test (RAVLT)) [111] were administered to study participants. Relationships between these clinical scales and the rs138397515 SNP are currently under investigation.

Our present work is a critical step in understanding the neurobiology of miR-20b and will propel other investigation in the near future. Our current focus was testing miR-20b’s primary target at mRNA and protein levels. The majority of the field simply investigates miRNA effects on mRNA, without reference to protein output. We further presented three interaction models (protein-protein, coexpression, and signaling network). Future work would, in addition to methods mentioned, include tracing downstream and parallel pathways through RNAseq, single-cell RNA assays, and proteomics, producing a fuller picture of miR-20b’s physiological roles. In a broader context, beyond miR-20b, this work should be expanded by roles of miRNA that may simultaneously function in AD and other disorders [112], influence or be influenced by currently-used drugs [7], non-pharmacological interventions, e.g., repeated electromagnetic field stimulation (REMFS) [5], and as a target or instigator of epigenetic modifications [2].

## Supplementary information

SUPPLEMENTAL MATERIAL
