# Supplementary material for: Human microRNA (miR-20b-5p) modulates Alzheimer’s disease pathways and neuronal function, and a specific polymorphism close to the MIR20B gene influences Alzheimer’s biomarkers
Source: Mol Psychiatry. 2022 Jan 27;27(2):1256–73. doi: 10.1038/s41380-021-01351-3 (PMC9054681; doi:10.1038/s41380-021-01351-3)
Supplement: Supplementary file 1 — SUPPLEMENTAL MATERIAL [file 41380_2021_1351_MOESM1_ESM.pdf]

**Human microRNA (miR-20b-5p) modulates Alzheimer's disease pathways and neuronal function, and a specific polymorphism close to the *MIR20B* gene influences Alzheimer's biomarkers.**

*Ruizhi Wang,*[1*] *Nipun Chopra,*[1*] *Kwangsik Nho,*[2*] *Bryan Maloney,*[1] *Alexander G. Obukhov,*[3] *Peter T. Nelson,* [4] *Scott E. Counts,*[5] *Debomoy K. Lahiri* [1,6#]

[1]Laboratory of Molecular Neurogenetics, Department of Psychiatry, Indiana Alzheimer's Disease Research Center, Indiana University School of Medicine, Indianapolis, IN-46202, USA;

[2]Radiology, Indiana University School of Medicine, Indianapolis, IN-46202, USA;

[3]Anatomy, Cell Biology & Physiology, Indiana University School of Medicine, Indianapolis, IN-46202, USA;

[4]Sanders-Brown Center on Aging, University of Kentucky, Kentucky Alzheimer's Disease Research Center, Lexington, KY, 40536, USA;

[5]Departments of Translational Neuroscience and Family Medicine, Michigan State University, Grand Rapids, MI, USA; Michigan Alzheimer's Disease Research Center, Ann Arbor, MI, 48109, USA

[6]Department of Medical & Molecular Genetics, Indiana University School of Medicine, Indianapolis, IN, USA;

*NC, RW and KN contributed equally

Present address: N. Chopra: DePauw University, Greencastle, IN 46135

# Corresponding author: dlahiri@iupui.edu

**Supplemental Table 1. Tjur's *D* of miR-20b vs. diagnosis models**

| Effect | Absolute Quantitation | | | | ΔΔCT | | | |
|---|---|---|---|---|---|---|---|---|
| | Full Model | TL[a] | CB | PCC | Full Model | TL | CB | PCC |
| miR-20b | 0.021 | 0.003 | 0.044 | < 0.001 | 0.024 | 0.117 | 0.048 | < 0.001 |
| *APOE*ε4 presence | 0.010 | 0.013 | 0.031 | < 0.001 | 0.085 | 0.191 | 0.098 | < 0.001 |
| Brain Region | 0.004 | 0.029 | < 0.001 | < 0.001 | 0.091 | 0.150 | 0.144 | < 0.001 |
| *APOE*ε4 × miR-20b | < 0.001 | 0.001 | 0.005 | < 0.001 | 0.020 | 0.110 | 0.039 | < 0.001 |

[a]TL, CB, and PCC refer to *D* calculated on the predictions of that brain region.

**Supplemental Table 2. Major AD-related proteins used as network building seeds.**

| Protein | Category[a] | miR-20b[b] |
|---|---|---|
| ADAM10 | Amyloid | + |
| ADAM17 | Amyloid | + |
| ADAM9 | Amyloid | + |
| APOE | Regulator | |
| APP | Amyloid | + |
| BACE1 | Amyloid | + |
| ECE1 | Clearance | |
| GSK3A | Tau | |
| GSK3B | Tau | |
| IDE | Clearance | + |
| IL1A | Amyloid | + |
| IRP1 | Amyloid | |
| IRP2 | Amyloid | |
| MAPK13 | Tau | |
| MAPT | Tau | + |
| MME | Clearance | + |
| PSD95 | Synaptic | |
| PSEN1 | Amyloid | |
| PSEN2 | Amyloid | |
| REST | Regulator | |
| SNAP25 | Synaptic | + |
| SNCA | Regulator | + |
| SYPH | Synaptic | |

[a]Classification vs. AD relationship, specifically, Amyloid: APP, APP processing enzyme, or APP translation factor; Clearance: Aβ clearing enzyme; Regulator: Protein with functions/effects on both Aβ and hyperphosphorylated tau protein; Tau: either MAPT or one of its major kinases.

bPredicted or confirmed to interact with miR-20b-50

**Supplemental Table 3. Predicted miR-20b-5p sites on VGCC subunits**

| **Subunit** | **Prob[a]** | **ΔG** |
|---|---|---|
| CACNA1A | no sites | no sites |
| CACNA1B | 0.148 | -17.4 |
| CACNA1C | 0.453 | -19.0 |
| CACNA1C | 0.386 | -16.9 |
| CACNA1C | 0.373 | -15.4 |
| CACNA1C | 0.305 | -17.7 |
| CACNA1D | 0.524 | -14.1 |
| CACNA1D | 0.431 | -20.1 |
| CACNA1E | 0.488 | -16.6 |
| CACNA1E | 0.298 | -14.7 |
| CACNA1F | no sites | no sites |
| CACNA1G | no sites | no sites |
| CACNA1H | no sites | no sites |
| CACNA1I | 0.125 | -24.3 |
| CACNA1I | 0.101 | -22.4 |
| CACNA1S | no sites | no sites |
| CACNA2D1 | 0.768 | -24.3 |
| CACNA2D1 | 0.604 | -15.8 |
| CACNA2D1 | 0.550 | -14.4 |
| CACNA2D1 | 0.375 | -13.8 |
| CACNA2D2 | 0.272 | -21.5 |
| CACNA2D3 | no sites | no sites |
| CACNA2D4 | 0.430 | -18.8 |
| CACNB1 | 0.272 | -16.6 |
| CACNB2 | 0.812 | -19.9 |
| CACNB2 | 0.463 | -21.0 |
| CACNB3 | no sites | no sites |
| CACNB4 | 0.840 | -19.8 |

[a]Estimated probability that the sequence affinity site is likely to be a miRNA binding site.

**Fig. S1. Treatment with miR-20b-5p and APP siRNA alters Calcium influx. A**) “Idealized” Fura response curve derived from mean of all traces. Specific points and distances of interest are described in main text. **B**) Raw mean Fura traces by treatment. **C**) Fura curves with values adjusted to the distance between “0” and “I” = 0. **D**) Fura curves scaled to minimum and maximum values for each treatment.

**Fig. S2. Typical photomicrographs of miR-20b treatment of PHB cultures.** PHB cultures were treated with miR-20b as described in the main text and grown in an incubator while photographed with the IncuCyte. Typical photographs for **A**) Mock-treated and **B**) miR-20b treated cells are shown. Apparent cell density reduction accompanied miR-20b treatment. Scales are shown on photographs.

**Fig. S3. Model of miR-20b-5p effects via APP on development and neurodegeneration.** A proposed model that relates our data to overall neurological effects on miR-20b-5p. **A**) Under developmental downregulation, increased miR-20b-5p would downregulate APP, resulting, through different pathways, of neurological pruning. **B**) If miR-20b-5p were upregulated, this would contribute to neuroproliferation. **C**) Late in life, elevation of miR-20b-5p would effectively enhance neuropreservation primarily through reducing excitotoxic processes. **D**) Reduction of miR-20b-5p late in life would contribute to excitotoxicity through APP upregulation, leading to neurodegeneration.

S1

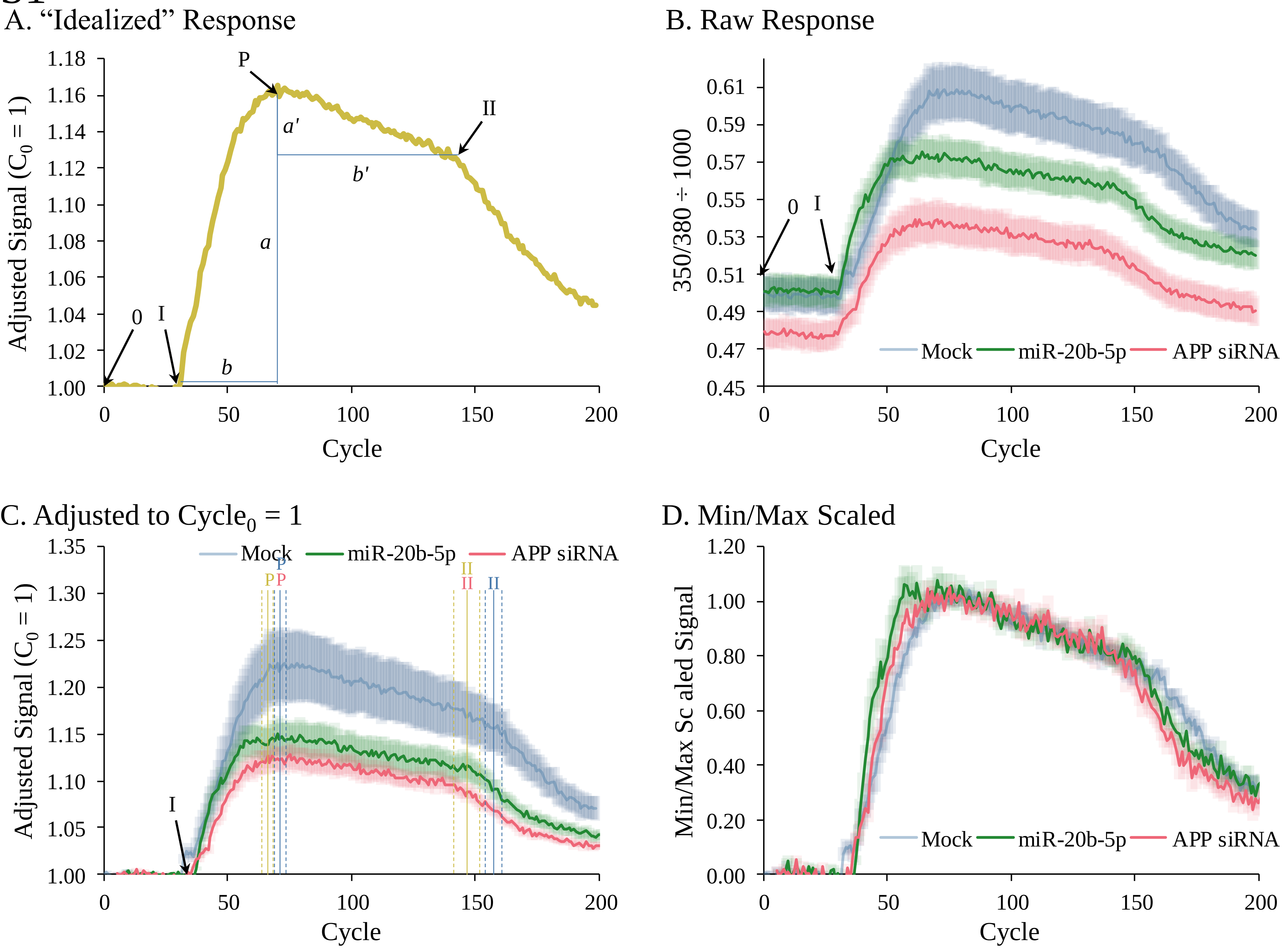

A. "Idealized" Response
Adjusted Signal ($C_0$ = 1)
P
II
a'
b'
a
b
0
I
Cycle
B. Raw Response
350/380 ÷ 1000
0
I
Mock
miR-20b-5p
APP siRNA
Cycle
C. Adjusted to Cycle$_0$ = 1
Adjusted Signal ($C_0$ = 1)
Mock
miR-20b-5p
APP siRNA
P
P
P
II
II
II
I
Cycle
D. Min/Max Scaled
Min/Max Sc aled Signal
Mock
miR-20b-5p
APP siRNA
Cycle

S2

A. Mock

B. miR-20b

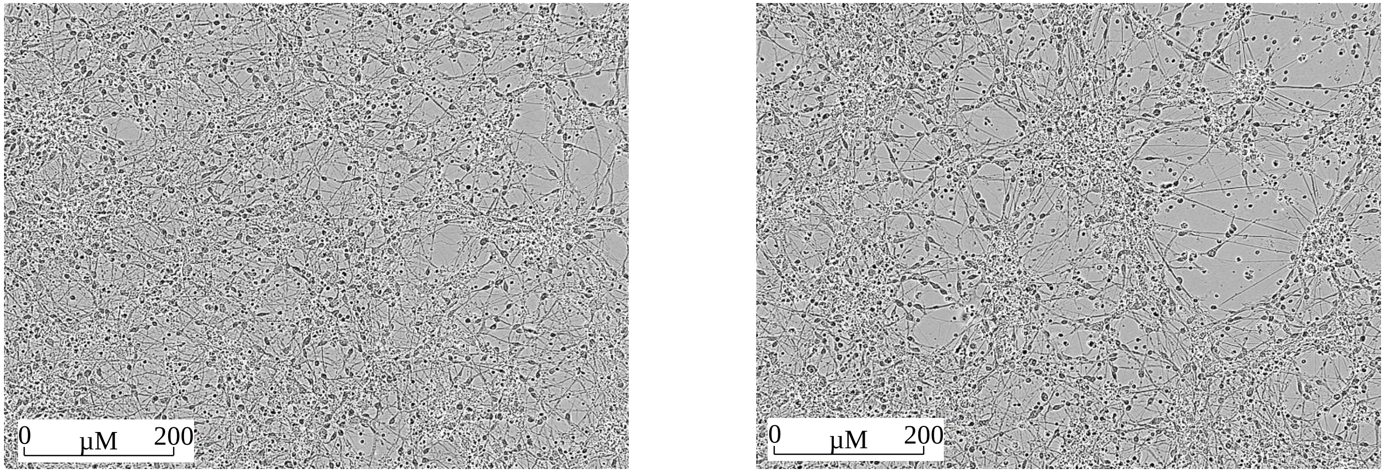

S3

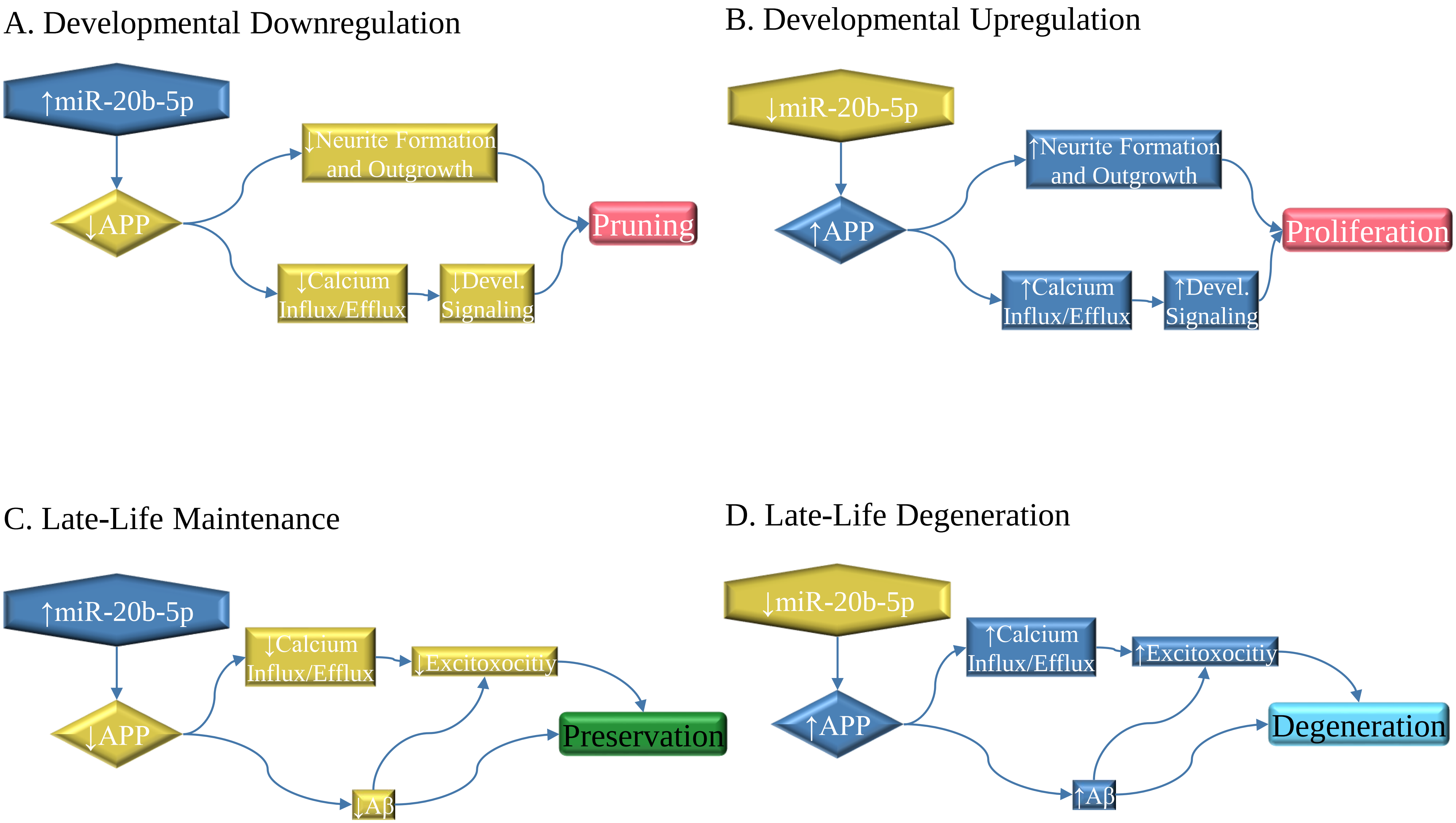

A. Developmental Downregulation
↑miR-20b-5p
↓APP
↓Neurite Formation and Outgrowth
↓Calcium Influx/Efflux
↓Devel. Signaling
Pruning
B. Developmental Upregulation
↓miR-20b-5p
↑APP
↑Neurite Formation and Outgrowth
↑Calcium Influx/Efflux
↑Devel. Signaling
Proliferation
C. Late-Life Maintenance
↑miR-20b-5p
↓APP
↓Calcium Influx/Efflux
↓Excitoxocitiy
↓Aβ
Preservation
D. Late-Life Degeneration
↓miR-20b-5p
↑APP
↑Calcium Influx/Efflux
↑Excitoxocitiy
↑Aβ
Degeneration
